# The Institutional Worker

**Published:** 1911-12-09

**Authors:** 


THE
### INSTITUTIONAL WORKER
**Being a Special Supplement to "The Hospital"**

### Editor's Notices.

**Contributions:**

Contributions should be written, or preferably typed, on one side of the paper only, and all articles sent in are accepted upon the distinct understanding that they are forwarded to The Hospital only.

The Editor cannot undertake to return MSS. not used, but when a stamped directed envelope is enclosed the MSS. may be returned if a special request is made.

Accepted articles and paragraphs of news will be paid for after publication at the scale rate.

**Address:**
To prevent delay all contributions and letters on editorial business must be addressed exclusively to the Editor, "The Hospital," 29 Southampton Street, Strand, London, W.C. It is important that this regulation shall be strictly observed.

**Correspondence:**

Correspondence on all subjects is invited. The name and address of correspondents must be given as a guarantee of good faith, but not necessarily for publication.

**Special Articles:**

Special articles are invited, and questions, inquiries, and paragraphs upon all matters relating to the work, administration, and management of general, special, mental, fever, cottage and convalescent hospitals, sanatoria, homes, institutions, societies and organisations for the treatment or care of the sick, injured and dependents of all classes. Every matter affecting the interests and welfare of the staff of all grades working in these institutions will receive special consideration.

**Administrative Medicine, Research and Items of News:**
Special terms will be paid for approved articles on subjects in this wide field by experts who have made some section of it their special study and interest. We wish to give prominence to every movement tending to advance accuracy of diagnosis, efficiency in treatment, and the development of modern methods to eradicate disease and promote the welfare of the suffering.

**A Bureau of Information:**
We invite inquiries and applications for information and help in providing for that numerous class of sufferers who require special care or house-room which they cannot obtain in their own homes or secure for themselves. We cannot, however, prescribe or recommend practitioners.

**Books for Review:**
Publishers are particularly requested to send advance proofs of any new books of importance whenever possible, as the Editor has made arrangements to publish immediate reviews on a new plan.

**Photographs, Plans, Blocks, and Illustrations:**
It is requested that wherever possible MSS. may be accompanied by illustrations in any of the above forms. The name of the sender and of the article to which it belongs should be written on the back of each photograph, drawing, or block for purposes of identification.

**Local Papers:**
Newspapers containing reports or news paragraphs should be marked and addressed to the Sub-Editor.

**The Coupon System:**
A coupon will be found at the bottom of the third inside page of the cover each week. This coupon must be attached to every question or inquiry to which an answer is desired in The Hospital.

### Manager's Notices.

Letters relating to the publication, sale, and advertising departments of The Hospital must be addressed to the Manager, "The Hospital," The Hospital Building, 28 and 29 Southampton Street, Strand, London, W.C.

**Subscriptions** which may begin at any time, are payable in advance. Cheques and Post-Office orders, crossed London County and Westminster Bank, Covent Garden branch, should be made payable to the Manager of The Hospital and addressed to him at The Hospital Building, 28 and 29 Southampton Street, Strand, London, W.C.

**Advertisements:**
All advertisements for The Hospital must reach the Manager not later than Wednesday morning in each week. For scale of charges see pages v and 2.

**Classified Advertisements:**
All small advertisements will be classified, for this section of the paper is widely read and of special interest. We wish to encourage those wanting appointments who are attracted to institutional work, and therefore offer them the reduced rate of *twenty-one words and under for* 1s., and *for every ten and part of ten words beyond,* 6*d.*

### POINTS FOR INSTITUTION AUTHORITIES.

The economical management of an Institution depends very greatly upon purchasing supplies in the cheapest market.

It is often impossible locally to secure the highest quality at the lowest prices.

In consequence, a section will be reserved in this part of the paper for announcements of Institutions inviting Tenders foı Contracts.

This will enable Authorities of Institutions to secure tenders for supplies from all over the country, and ensure purchases at the lowest possible prices consistent with quality.

The charge for Tenders is 6s. per inch, and for this small outlay many pounds may be saved on supplies of all descriptions.

**Special Rates** will be quoted for those institutions which agree to send all their vacancy, contract, and public notices to The Hospital each year, so as to make it a file of such announcements for ready reference.

### POINTS FOR TRADE ADVERTISERS.

The Hospital is the organ of Institutions, whether of a Voluntary Character or those under Local Government, Poor Law and Municipal Authorities.

It is read by all Executive Officials and those responsible for the Construction, Management, and Equipment of Public Establishments.

It is also the Journal of the worker engaged in the Medical, Health and Preventive services.

There is no medium more appropriate and effective in the promotion of business in these directions than The Hospital.

### POINTS FOR INSTITUTIONAL WORKERS.

A very large number of Appointments Vacant appear every week in the columns of The Hospital.

If you cannot find a position suited to your requirements, make your wishes known through our Situations Wanted Column.

### APPOINTMENTS VACANT.

### Poor-Law Vacancies.

### Appointments Vacant.

Assistant Female Relieving Officer (£75), Norwich Guardians. Mr. E. J. W. Huggins, C. to G., Norwich.

Assistant Male Attendant (£30), Southampton Union. Mr. A. J. Walden, C. to G., Southampton. Dec. 9.

Assistant Matron (£40), Southampton Union. Mr. A. J. Walden, C. to G., Southampton. Dec. 16.

Assistant Matron (£25), Whitehaven Union. Mr. J. Bowly, C. to G., Whitehaven. Dec. 18.

Assistant Matron (£25), Hartley Wintney Union. Mr. W. H. Wright, C. to G., Odiham, Hants.

Assistant Nurse (£22), Bermondsey Guardians. E. Pitts Fenton, C. to G., 283 Tooley Street, S.E. Dec. 11.

Assistant Nurse (£25). Newbury Union. S. V. Pinniger, C. to G., Market Place, Newbury. Dec. 15.

Assistant Nurse (£20), Kingsclere Union. J. Barnes, C. to G., Kingsclere, Newbury.

Assistant Nurse (£25), Pontypridd Union. Mr. W. Spickett, C. to G., Pontypridd. Dec. 12.

Assistant Nurses (£25), St. Thomas Union. Mr. W. B. Trick, C. to G., St. Thomas, Exeter. Dec. 14.

Assistant Nurse (£25), Whitehaven Union. Mr. J. Bowly, C. to G., Whitehaven. Dec. 18.

Assistant Nurse (£25), Wortley Union. Mr. J. Morton, C. to G., Union Offices, Grenoside, Sheffield.

Charge Nurse (£30), Haslingden Union. J. H. Sinkinson, C. to G., Union Offices, Rawtenstall. Dec. 12.

Charge Nurse (£30), South Shields Union. J. W. Coulson, C. to G., Barrington Street, S. Shields. Dec. 16.

Charge Nurses (£24), City of London Union. Matron, City of London Infirmary, Clifden Road, N.E.

Charge Nurse (£32), Cheltenham Union. Mr. J. Meek, C. to G., Swindon Road, Cheltenham. Dec. 20.

Children's Trainer (£23), Preston Union. Mr. J. Clarke, C. to G., Preston. Dec. 14.

Female Teacher (£70), Toxteth Park Guardians. Mr. R. A. Janes, C. to G., High Park Street, Liverpool. Dec. 13.

Foster Mother (£22), Hull Guardians. Mr. R. H. Winter, C. to G., Hull. Dec. 12.

Industrial Trainer and Foster-Mother (married couple) (£35 and £30), Kingston Union. Mr. C. W. Dash, C. to G., Kingston-on-Thames.

Junior Assistant Nurses (£15), Bishop's Stortford Union. A. G. Gwynn, C. to G., Bishop's Stortford. Dec. 9.

Labour Master (£40), Kidderminster Union. Mr. F. E. Burcher, C. to G., Kidderminster. Dec. 9.

Labour Master (£30), Hartley Wintney Union. Mr. W. H. Wright, C. to G., Odiham, Hants.

Labour Master (£24), Dore Union. Mr. R. Moore, C. to G., Hereford. Dec. 9.

Male Attendant (£26), Islington Guardians. Master of the Workhouse, St. John's Road, Upper Holloway, N.

Male Night Nurse (27s. weekly, non-resident). Mr. A. J. Harris, C. to G., Cardiff. Dec. 11.

Master and Matron (£80 and £50), Carlisle Union. Mr. H. B. Lonsdale, C. to G., Carlisle. Dec. 11.

Master and Matron (£50 and £30), Penistone Union. Mr. C. Hodgkinson, C. to G., Penistone. Dec. 13.

Master and Matron (£100), Grimsby Union. Mr. J. F. Wintringham, C. to G., Great Grimsby. Jan. 1.

Nurse (£30), Fylde Union. F. H. Browne, C. to G., Union Offices, Wesham, Kirkham. Dec. 12.

Nurse (£30), Ticehurst Union. J. Chase Edwards, C. to G., Union Offices, Wadhurst, Sussex. Dec. 16.

Nurse (£25), Blean Union. Mr. J. E. Burch, C. to G., 39 Castle Street, Canterbury. Dec. 11.

Nurse (£26), Chesterton Union. Mr. J. F. Symonds, C. to G., Cambridge. Dec. 15.

Nurse (£25), Dursley Union. Mr. J. G. Wenden, C. to G., Union Offices, Dursley. Dec. 14.

Probationer (£12), Isle of Wight Union. Mr. A. G. Harrison, C. to G., Newport, I.W. Dec. 12.

Probationers (£13), West Ham Union. Mr. T. Smith, C. to G., Union Road, Leytonstone.

Schoolmaster (£65), West Ham Union. Mr. T. Smith, C. to G., Leytonstone, N.E. Dec. 20.

Sister (£28), Paddington Guardians. Matron of the Infirmary, 285 Harrow Road, W.

Superintendent Nurse (£40), Windsor Union. Mr. P. Lovegrove, C. to G., 3 Park Street, Windsor. Dec. 26.

Tailoring Attendant (£35), St. Pancras Guardians. Mr. J. E. P. Hall, C. to G., Town Hall, Pancras Road, N.W.

Temporary Infant Protection Visitor (£2 2s. weekly), Edmonton Union. Mr. F. Shelton, C. to G., White Hart Lane, Tottenham. Dec. 9.

Temporary Staff Nurses (3) (£20), Holborn Union. Matron of the Infirmary, Archway Road, Upper Holloway, N.

Ward Sister (£30), Hammersmith Guardians. C. to G., 206 Goldhawk Road, Shepherd's Bush. Dec. 11.

Ward Sister (£30), Southampton Union. Mr. A. J. Walden, C. to G., Southampton. Dec. 16.

Ward Sister (£30), St. Pancras Guardians. Mr. J. E. P. Hall, C. to G., Town Hall, Pancras Road, N.W.

### Situations Vacant.

Assistant Cook (£20), Hammersmith Guardians. The C. to G., Goldhawk Road, Shepherd's Bush. Dec. 9.

Assistant Laundress (£24), Bath Union. Mr. W. E. Winckworth, C. to G., Bath. Dec. 9.

Assistant Laundress (£20), Paddington Guardians. Mr. F. J. P. Jordan, acting C. to G., 313-319 Harrow Road, W. Dec. 13.

Bathman and Barber (£25), Hunslet Union. Mr. F. W. Mee, C. to G., Hunslet. Dec. 13.

Cook (£25), Settle Union. Mr. T. E. Pearson, C. to G., Settle. Dec. 11.

Cook-General (£23), Eastbourne Union. The Matron of the Workhouse, Eastbourne.

Female Cook (£28), Winchester Union. Mr. F. Faithfull, C. to G., Winchester. Dec. 11.

Female Cook (£30), Ticehurst Union. Mr. J. C. L. Andrews, C. to G., Wadhurst. Dec. 16.

Female Cook (£25), Bosmere and Claydon Union. The Matron of the Workhouse, Barham, near Ipswich.

Hospital Cook (£27), Burton-on-Trent Union. Mr. C. F. Chamberlin, C. to G., Burton-on-Trent. Dec. 18.

Laundry Checker, Bolton Union. Mr. H. I. Cooper, C. to G., Bolton. Dec. 9.

Porter (£28), King's Lynn Union. Mr. R. C. Coulton, C. to G., King's Lynn. Dec. 12.

Porter, Bolton Union. Mr. H. I. Cooper, C. to G., Bolton. Dec. 9.

Porter, &c. (£30), Stratford-on-Avon Union. Mr. S. C. Warden, C. to G., Stratford-on-Avon. Dec. 9.

Porter (£30), Newbury Union. Mr. S. V. Pinniger, C. to G., Newbury. Dec. 13.

Porter and Handy Man (£25), Bridlington Union. Mr. G. Hankinson, C. to G., Bridlington. Dec. 12.

Second Assistant Laundress (£25), St. Pancras Guardians. Mr. J. E. P. Hall, C. to G., Town Hall, Pancras Road, N.W. Dec. 9.

### Institutional and Administrative Vacancies.

### Appointments Vacant.

Matron (£150), Anglo-American Hospital, Cairo, Egypt. Secretary. Dec. 20.

Matron (£45), Romsey Nursing Home and Cottage Hospital. Hon. Secretary.

Night Superintendent (£37), Salop Infirmary, Shrewsbury. Apply Matron.

Staff Nurse (£28), Eston Hospital, Middlesbrough. Apply Matron.

### Municipal Vacancies.

### Appointments Vacant.

Assistant Inspector of Weights and Measures (£100), Burnley T.C. Mr. P. Thomas, Town Clerk, Burnley. Dec. 11.

Assistant Nurses (£24), Manchester Corporation. Matron Baguley Sanatorium, Timperley, Cheshire.

Charge Nurse (£35), Borough of Southampton Isolation Hospital. Medical Officer of Health, Municipal Offices, Southampton.

County Surveyor's Clerk (£90), Cornwall C.C. Mr. A. E. Brookes, County Surveyor, Truro. Dec. 9.

Engineer for Sewage Disposal Works (£2 weekly), Walsall T.C. Mr. J. R. Cooper, Town Clerk, Walsall. Dec. 11.

Health Visitor (£75), Great Yarmouth Town Council. Mr. W. E. Stephens, Town Clerk. Dec. 12.

Health Visitor (£75), Borough of Chesterfield. Mr. J. Middleton, Town Clerk, Chesterfield. Dec. 16.

Lady Health Visitor (£75), City of Wakefield. Mr. W. W. Greenhalgh, Town Clerk. Dec. 12.

Matron (£60), Florence Nightingale Hospital, Bury. The Clerk, District Joint Hospital Board, Cross Street, Bury, Lancs. Dec. 12.

Nurse (£26), Llandaff and Dinas Powis Rural District Council Isolation Hospital. Mr. M. Warren, Clerk, Park House, Cardiff. Dec. 27.

Probationers (£15), Manchester Corporation. Matron, Baguley Sanatorium, Timperley, Cheshire.

Staff Nurses (£26), Manchester Corporation. Matron, Baguley Sanatorium, Timperley, Cheshire.

### Miscellaneous Vacancies.

Attendants (married couple) (£78 joint), Weymouth Port Sanitary Authority. Mr. J. J. Roberts, Clerk to the Authority, St. Thomas Street, Weymouth. Dec. 14.

Matron (£50), Bolton Education Committee, Women's Training College Hostel. Mr. F. Wilkinson, Education Offices, Bolton. Dec. 9.

Nurses (£19 10s.), Colney Hatch Asylum. Medical Superintendent, New Southgate, N.

School Nurse (£70), City of York Education Committee. Mr. J. H. Mason, Secretary, Education Offices, Clifford Street, York. Dec. 27.

School Nurse (£75), Essex Education Committeee. Mr. J. H. Nicholas, Secretary, County Offices, Chelmsford. Dec. 15.

Veterinary Inspector, Wilts C.C. Mr. R. W. Merriman, Clerk to the Council, Trowbridge. Dec. 31.

### APPOINTMENTS, RESIGNATIONS, &c.

### Poor Law Appointments.

Hall, Mr. C. W. J., Shoreditch Parlsh, Junior Assistant Clerk.

Laing, Mr. C. Y., South Manchester Township, Junior Resident Assistant Medical Officer at the Infirmary.

McPhail, Mr. J. M., Bristol Parish, Resident Assistant Medical Officer for the Workhouses and Head Quarters Homes.

Thompson, Mr. C. C., Doncaster Union, Relieving Officer.

Townsend, Mr. J., Selby Union, Assistant Clerk.

Masters and Matrons.

Bareham, Mr. R., Burton-upon-Trent Union.

Bareham, Mrs. R., Burton-upon-Trent Union.

Benny, Mr. R. H., Bodmin Union.

Benny, Mrs. M. E., Bodmin Union.

